# Severe dengue, aneurysmal sub-arachnoid hemorrhage, and hemophagocytic lymphohistiocytosis: a rare case combination

**DOI:** 10.31744/einstein_journal/2025RC1209

**Published:** 2025-02-14

**Authors:** Saboor Mateen, Ajay Mishra, Shivesh Singh, Firdaus Jabeen

**Affiliations:** 1 Era’s Lucknow Medical College & Hospital Lucknow Uttar Pradesh India Era’s Lucknow Medical College & Hospital, Lucknow, Uttar Pradesh, India.

**Keywords:** Dengue, Severe dengue, Fever, Lymphohistiocytosis, hemophagocytic, Haemorrhage, Subarachnoid hemorrhage

## Abstract

Dengue, a vector-borne acute febrile illness caused by members of the *Flavivirus* genus, has dramatically increased its occurrence worldwide. Neurological complications of dengue range from 2.63 to 40%, and subarachnoid hemorrhage is a rare, but significant manifestation. Hemophagocytic lymphohistiocytosis is a life-threatening hyperinflammatory syndrome, sometimes secondary to infections such as dengue. This report presents a rare case of severe dengue with subarachnoid hemorrhage and hemophagocytic lymphohistiocytosis. A 19-year-old male presented with a 7-day history of fever and myalgia, followed by severe headache and vomiting. Initial examination revealed high fever, hepatosplenomegaly, and pancytopenia. Lumbar puncture confirmed via computed tomography showed a Fisher Grade 2 subarachnoid hemorrhage with a small aneurysm at the junction of the left anterior coronary and anterior communicating arteries. Secondary hemophagocytic lymphohistiocytosis was diagnosed based on the criteria from 2004, with elevated inflammatory markers, hypertriglyceridemia, and hyperferritinemia. The patient was treated conservatively with intravenous fluids, osmotic diuretics, antiepileptics, steroids, and nimodipine. The patient showed clinical improvement and was discharged on the 11^th^ day. Isolated subarachnoid hemorrhage is rare in dengue. The hyperinflammatory state in hemophagocytic lymphohistiocytosis, which is often overlooked due to nonspecific symptoms, can lead to aneurysm formation and rupture. Persistent fever, cytopenia, and hyperferritinemia should raise suspicion of hemophagocytic lymphohistiocytosis in cases of severe dengue with neurological complications. In patients with severe dengue and intracranial hemorrhage, clinicians should remain cautious for hemophagocytic lymphohistiocytosis to reduce the associated morbidity and mortality.

## INTRODUCTION

Dengue, a vector-borne acute febrile illness, is caused by members of the *Flavivirus* genus and has spread dramatically worldwide in recent decades, with cases reported by the World Health Organization escalating from less than 1 million in 2000 to 5.2 million in 2019.^1^ India is among the top five endemic areas in Southeast Asia, contributing significantly to the global burden of dengue.^2^ The neurological complications of dengue vary between 2.63-40%, most commonly including encephalopathy, encephalitis, and syncope, followed by acute symptomatic seizures, Guillain-Barre syndrome, and intracranial hemorrhage.^3^

Subarachnoid hemorrhage (SAH) can occur secondary to dengue infection, the pathophysiology of which is multifactorial and attributed to the complex interplay of vasculopathy, coagulopathy, platelet dysfunction, and thrombocytopenia.^4^ Hemophagocytic lymphohistiocytosis (HLH) or hemophagocytic syndrome is an emergent, potentially life-threatening, hyperinflammatory response syndrome caused by a large cytokine storm that can be genetic (familial/primary) or acquired (secondary/reactive), with the latter including dengue.^5^ Subarachnoid hemorrhage has been previously reported in HLH, occurring as a late effect following treatment or as an autopsy finding.^6^

Although isolated SAH in dengue and HLH has been noted separately, HLH secondary to dengue with isolated SAH has not been reported. We present the case of a young male with severe dengue fever who developed SAH against the backdrop of HLH.

## CASE REPORT

A 19-year-old male presented with complaints of fever with myalgia for 7 days, along with severe headache and persistent vomiting for 1 day prior to admission. Upon initial assessment, he was conscious and alert, with a pulse rate of 106 bpm, blood pressure of 110/70mmHg, temperature of 103 °F, respiratory rate of 26 breaths/min, and a blood oxygen saturation of 98% while breathing ambient air (room air, without supplemental oxygen). The results of the remaining general examinations were unremarkable. Upon physical examination, central nervous system assessment, including the fundus, was normal, with a Glasgow Coma Scale score of 15/15 and no focal neurological *deficit*. An abdominal examination revealed hepatosplenomegaly with no signs of free fluid. Cardiovascular and respiratory system evaluations were unremarkable.

The hematological profile showed pancytopenia with a reduced absolute neutrophil count and low hematocrit. The coagulation profile was within normal limits. The biochemical profile revealed elevated serum transaminase, lactate dehydrogenase, and ferritin*.* The lipid profile revealed high triglyceride and very low-density lipoprotein*.* The remaining biochemical profiles, including renal function, electrolytes, and thyroid function tests, were within normal limits *(*Table 1*)*. The urine and blood culture results were sterile. ECG and chest radiographic findings were normal. Abdominal ultrasonography revealed hepatosplenomegaly with calculus cholecystitis but no signs of free fluid formation. Based on the clinical symptoms (fever, myalgia, headache, and vomiting), signs (palpable hepatosplenomegaly), and laboratory findings (pancytopenia with deranged liver function tests), dengue fever was suspected in the context of endemicity and seasonality.^2^ It was subsequently confirmed through positive anti-dengue IgM and IgG antibodies in ELISA. The patient was diagnosed as a case of confirmed dengue fever with warning signs.^7^

**Table 1:** Lab investigations

Parameter	Lab-value		Range
	Day 1	Day 6	
Complete blood count			
Hemoglobin (g/dl)	9.4 g/dL	10.5 g/dL	10-16.5 g/dL
Total leukocyte count (cells/cumm)	2,400 cells/cumm	8,400 cells/cumm	4,000-11,000 cells/cumm
Differential leukocyte count - neutrophil percentage (%)	35	54	40-75
Absolute neutrophil count (cells/µL)	840 cells/µL	4,536 cells/µL	2,500-6,000 cells/µL)
Platelet count (x109/L)	30 x 109/L	210 x 109/L	150 to 400 x 109/L
Prothrombin time (s)	12.4	11.4	10-13
International normalized ratio	1.10	1.04	0.6-1.5
Hematocrit (%)	29.1	35.1	33-54
Kidney function tests			
Blood urea (mg/dL)	30 mg/dL	28 mg/dL	Female: 15-36 mg/dL
Creatinine (mg/dL)	1.0 mg/dL	0.8 mg/dL	Female: 0.7-1.2 mg/dL
Serum sodium (mmol/L)	140 mmol/L	145 mmol/L	135-45 mmol/L
Serum potassium (mmol/L)	4.4 mmol/L	4.0 mmol/L	3.5-5.5 mmol/L
Liver function tests			
Serum bilirubin (total) (mg/dL)	1.3 mg/dL	1.0 mg/dL	0.2-1.3 mg/dL
Direct bilirubin (mg/dL)	0.2 mg/dL	0.3 mg/dL	0.0-0.3 mg/dL
Indirect bilirubin (mg/dL)	1.1 mg/dL	0.7 mg/dL	0.2-0.8 mg/dL
Alanine transaminase (U/L)	186 U/L	103 U/L	Men: ≤45 U/L
Aspartate aminotransferase (U/L)	276 U/L	139 U/L	Men: up to 35 U/L
Serum alkaline phosphatase (U/L)	252 U/L	104 U/L	38-125 U/L
Others			
Serum calcium (mg/mL)	8.1 mg/dL	9.0 mg/dL	8.6-10.2 mg/dL
Total protein (g/dL)	5.6 g/dL	-	Adult: 6.4-8.3 g/dL
Serum albumin (g/dL)	3.2 g/dL	3.6 g/dL	Adult: 3.5-5.2 g/dL
Serum lactate dehydrogenase (U/L)	473 U/L	318 U/L	120 - 246 U/L
Serum ferritin (ng/mL)	4500 ng/mL	656 ng/mL	Male: 17.9–464 ng/mL
Serum C-reactive protein (mg/L)	>90 mg/L	8 mg/L	<5 mg/L
Lipid profile			
Serum cholesterol (mg/dL)	160 mg/dL	156 mg/dL	Borderline high: 200–239 mg/dL
Serum triglyceride (mg/dL)	394 mg/dL	388 mg/dL	<161.0 mg/dL
Serum high-density lipoprotein (mg/dL)	27 mg/mL	27 mg/mL	Male: 35.3-79.3 mg/dL
Serum very low-density lipoprotein (mg/dL)	65 mg/dL	67 mg/dL	2-30 mg/dL
Serum low-density lipoprotein (mg/dL)	69 mg/dL	72 mg/dL	≤100 mg/dL
Cerebrospinal fluid analysis			
Opening pressure	35 cmH_2_O	-	6-25 cmH_2_O
Color/cells	Red with fair number of red blood cells	-	Clear and colorless
Total leukocyte count (cells/cumm)	35 cells/mm^3^	-	<5 cells/mm^3^
Protein	208 mg/dL	-	12-60 mg/dL
Sugars	76 mg/dL	-	45-60 mg/dL

Lumbar puncture was performed, which revealed red cerebrospinal fluid, with increased opening pressure and increased total cell count, proteins, and sugars (Table 1).

**Figure 1:**
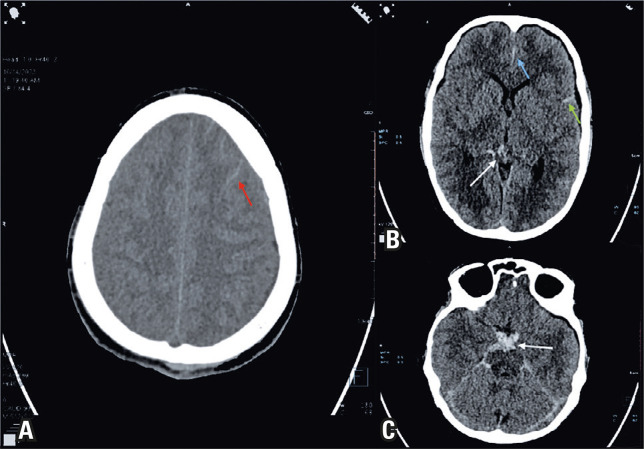
Acute subarachnoid hemorrhage in the (A) left frontal sulci (red arrow), (B) left Sylvian fissure (green arrow) and anterior inter hemispheric fissure (blue arrow), and (C) suprasellar (white arrow), basal, peri-mesencepahlic, and prepontine cisterns (B- white arrow)

**Figure 2:**
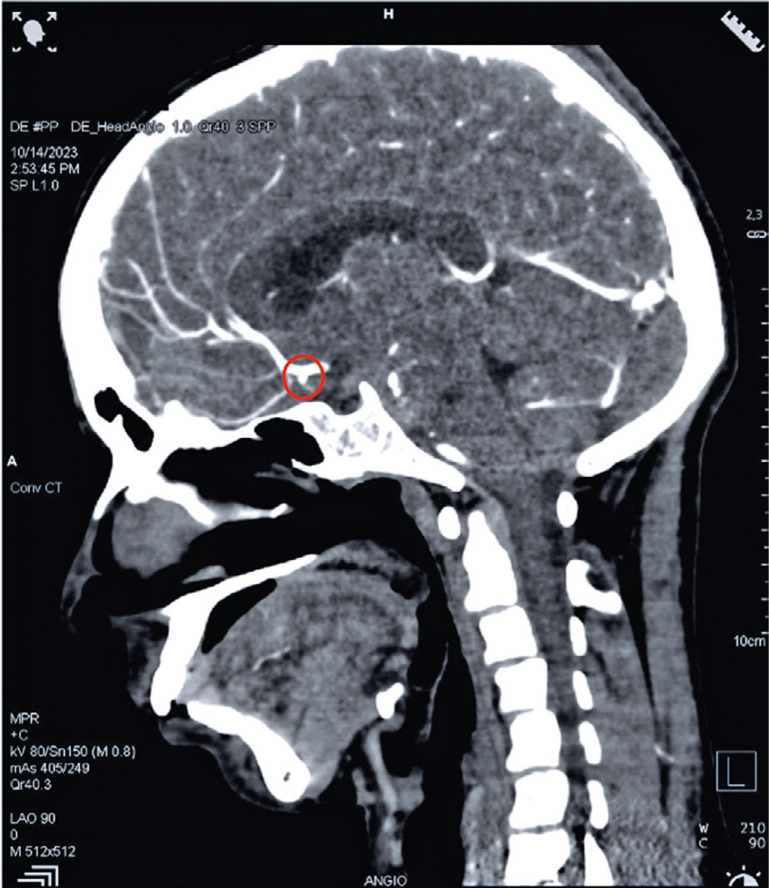
Head CT (sagittal view) of an outpouching left of the ACA-ACom junction (red circle)

**Figure 3:**
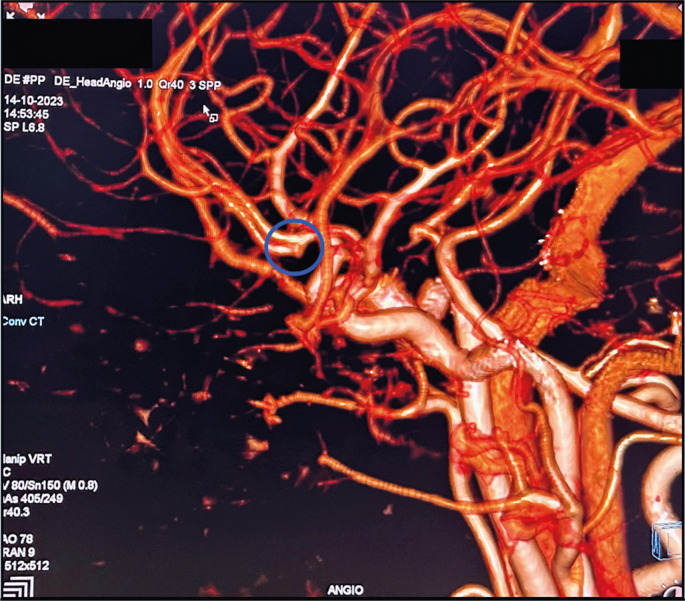
CTCA 3D reformation of a beaded outpouching at the anterior coronary-anterior communicating artery junction (blue circle)

Non-contrast computed tomography (CT) of the head indicated acute Fisher Grade 2 SAH along the left frontal sulci, Sylvian fissure, and anterior interhemispheric fissure (Figure 1). CT cerebral angiography (CTCA) showed a small outpouching at the left anterior coronary artery (ACA)-anterior communicating artery (ACom) junction measuring approximately 1.5 mm (Figures 2 and 3). Bone marrow examination was not performed, as it is a stressful procedure, and was avoided to prevent re-bleeding in the context of an already identified intracranial aneurysm, adhering to the principle of beneficence.

Considering the elevated inflammatory mediators and pancytopenia with no signs of plasma or fluid leakage, secondary HLH was suspected. This diagnosis was confirmed based on the HLH-2004 clinical criteria,^8^ which comprise molecular and non-molecular endpoints, at least five of eight of which are needed to diagnose HLH. The patient fulfilled the following criteria: high-grade fever for at least 7 days, splenomegaly, bicytopenia, hypertriglyceridemia, and hyperferritinemia. The H-score^8^ was 229, which showed a probability of 96-98% for HLH. The final diagnosis was severe dengue with the possibility of secondary HLH with isolated SAH.

The patient was treated conservatively with intravenous fluids in the form of crystalloids (0.9% normal saline) that were initially started at 20 mL/kg for the first 1 h of admission, followed by a gradual decrease to 10 mL/kg for the next 6 h and a maintenance rate of 5 mL/kg for the next 48 h. A calcium channel blocker, nimodipine tablet, was administered at 60 mg every 4 h for 21 days. Oral acetaminophen (650 mg) was administered for 8 h for 4 days to treat the headaches. Osmotic diuretics in the form of 20% intravenous mannitol (20 g) were administered every 6 h for 2 days, every 12 h for the next 2 days, and then stopped. Antiepileptics in the form of oral levetiracetam were administered at 1 g for 12 h for the first 7 days, followed by 500 mg every 12 h for the next 3 weeks. Oral dexamethasone was commenced at 10 mg/m^2^/day in two divided doses for two weeks, followed by a reduction in dosing of half strength every two weeks for the next six weeks. Etoposide injection was administered at 300 mg twice per week for the first 2 weeks, followed by one dose weekly for the next 6 weeks. The patient improved clinically, with a reduction in headache intensity over the next five days, along with the resolution of thrombocytopenia and acute-phase reactants (Table 1). The patient was discharged on the 11^th^ day of illness and advised to undergo follow-up. This study was approved by the Institutional Research Ethics Committee (Registration No. ECR/717/ Inst. / UP/ 2015/ RR-21.

## DISCUSSION

Severe dengue involves dengue fever accompanied by a range of symptoms and sequelae, including plasma leakage, severe bleeding, and significant multiorgan involvement.^9,10^ The present case involved fever, severe headache, and persistent vomiting-common symptoms observed during the febrile phase of dengue fever,^9,10^ which concomitantly progressed to the critical phase, as evidenced by severe hemorrhagic manifestations.

Headache associated with dengue is typically severe, presenting as frontal and retro-ocular pain, and is one of the most prevalent symptoms, occurring in up to 97.6% of cases, as reported by Domingues et al.^11^ In contrast, headache resulting from a ruptured aneurysm in SAH is particularly deadly, with a median case-fatality rate of 27-44%.^12^ It is described as a “thunderclap headache,” characterized by sudden onset and severe intensity, often considered the worst headache the patient has ever experienced. The patient exhibited an acute-onset headache that was persistent and agonizing, prompting us to evaluate differential diagnoses, including SAH. Therefore, recognizing the presence and pattern of severe headaches is crucial for raising the suspicion of dengue fever.

Although intracranial hemorrhages, including SAH, have been reported in patients with severe dengue, isolated SAH in the context of dengue rarely has been discussed.^4,13,14^ In our case, the absence of bleeding manifestations other than SAH raised the possibility of cranial pathologies, such as an arteriovenous malformation or aneurysm, as the cause of isolated SAH. Given that saccular unruptured intracranial aneurysms are found in approximately 3% of the adult population^15^ and are the most common incidental findings on neuroimaging in healthy individuals,^16^ CTCA was performed, which confirmed the presence of an aneurysm.

Both primary and secondary HLH present with a wide array of nonspecific clinical symptoms that are often overlooked owing to similarities with sepsis and other hyperinflammatory conditions. Persistent fever despite antibacterial therapy, negative cultures, cytopenia, hyperferritinemia, and multisystem involvement should raise suspicion of HLH, as outlined in a recent review by See.^17^

Studies have explored the role of hyper-inflammatory states in the formation, growth, and rupture of aneurysms.^18^ A critical unanswered question of our case is whether the aneurysm was a preexisting benign condition or a consequence of the hyperinflammatory state associated with HLH.

Treatment in these cases is challenging and varies significantly based on the nature and extent of the bleeding, as well as the overall patient condition and progress. We opted for conservative management, which was successful and aligned with the findings of a case series by Sam et al.^13^ A combination of high-dose steroids, osmotic diuretics, calcium channel blockers, and tailored fluid management can effectively address hyper-inflammatory states associated with intracranial bleeding in dengue.

Aneurysmal rupture during a hyperinflammatory process, such as HLH, initiated by hemodynamic changes in severe dengue, may result from matrix metalloproteinase (MMP)-mediated degradation of the extracellular matrix and apoptosis of smooth muscle cells (SMCs). These processes progressively weaken the arterial wall, leading to dilation, aneurysm formation, and rupture.^18^

Macrophages and SMCs are the two main constituents of the inflammatory and associated degenerative responses. The former are invariably present in cerebral aneurysms,^17^ whereas the latter release MMPs. The duality of macrophage-associated aneurysmal maturation and rupture has also been shown by Aoki et al.,^19^ who revealed that macrophages and macrophage-derived MMPs are linked to aneurysm growth, and that SMC proliferation and macrophages are highly associated with aneurysmal rupture.

## CONCLUSION

In patients with severe dengue and associated intracranial hemorrhage, a high index of suspicion should be maintained for hyperinflammatory conditions, such as hemophagocytic lymphohistiocytosis, which can help to reduce the associated mortality and morbidity.
